# Synthesis and Electrophilic Substitutions of Novel Pyrazolo[1,5-*c*]-1,2,4-triazolo[4,3-*a*]pyrimidines

**DOI:** 10.3390/molecules16087081

**Published:** 2011-08-18

**Authors:** Kamal F.M. Atta

**Affiliations:** Chemistry Department, Faculty of Science, Alexandria University, Ibrahimia P.O. Box 426, Alexandria 21321, Egypt; Email: prof.kf_atta@yahoo.com; Tel.: 002035917883; Fax: 002035932488

**Keywords:** pyrazolo[1,5-*c*]-1,2,4-triazolo[4,3-*a*]pyrimidines, synthesis, electrophilic substitutionreactions, dehydrative cyclization

## Abstract

5-Aryl-7-hydrazino-2-phenylpyrazolo[1,5-*c*]pyrimidines **1** were used as precursors for the preparation of a new series of 5-aryl-8-phenylpyrazolo[1,5-*c*]-1,2,4- triazolo[4,3-*a*]pyrimidines **2**. The reactions of **2** with certain electrophilic reagents gave the respective 6-substituted derivatives **3**-**5** rather than the 7-isomeric products. Formylation of the key compounds **1** with ethyl formate yielded the formyl derivatives **6**. Furthermore, boiling of compounds **1** with acetic acid afforded 7-acetylhydrazino-5-aryl-2-phenylpyrazolo[1,5-*c*]pyrimidines **7**. Bromination of **7** yielded the dibromo- derivatives **8**, while their iodination and nitration gave the monosubstituted derivatives **9** and **10**, respectively. Also, treatment of **1** with boiling acetic anhydride yielded the triacetyl derivatives **11**. The structure of synthesized products was confirmed by elemental analyses, IR, ^1^H NMR and MS spectra.

## 1. Introduction

The pyrazolo[1,5-*c*]pyrimidine ring represents a biologically and synthetically important class of compounds. Many pyrazolo[1,5-*c*]pyrimidines are known to possess significant hypnotic, tranquilizing, fungicidal, insecticidal and antibacterial activities [1,2,3]. Also, the coordination of pyrazolo[1,5-*c*]pyrimidines to transition metal ions such as Cu^+2^ and Ni^+2^ enhances their biological activities [4,5,6,7].

In the last few decades, the chemistry of 1,2,4-triazoles and their fused heterocyclic derivatives has received considerable attention owing to their synthetic and effective biological importance. 1,2,4- Triazole moieties have been incorporated into a wide variety of therapeutically interesting drug candidates, e.g., triazolam [8], alprazolam [9], etizolam [10], and furacylin [11], including anti- inflammatories, central nervous system stimulants, sedatives, anti-anxiety compounds, antimicrobial agents [12,13,14,15] and antimycotic ones such as fluconazole, intraconazole, voriconazole [16,17].

The above mentioned therapeutic activity has prompted the present investigation to synthesize the pyrazolo[1,5-*c*]-1,2,4-triazolo[4,3-*a*]pyrimidines ring system **2** and a new series of 7-acetylhydrazino-5-aryl-2-phenylpyrazolo[1,5-*c*]pyrimidine derivatives **7**.

## 2. Results and Discussion

**Scheme 1:**
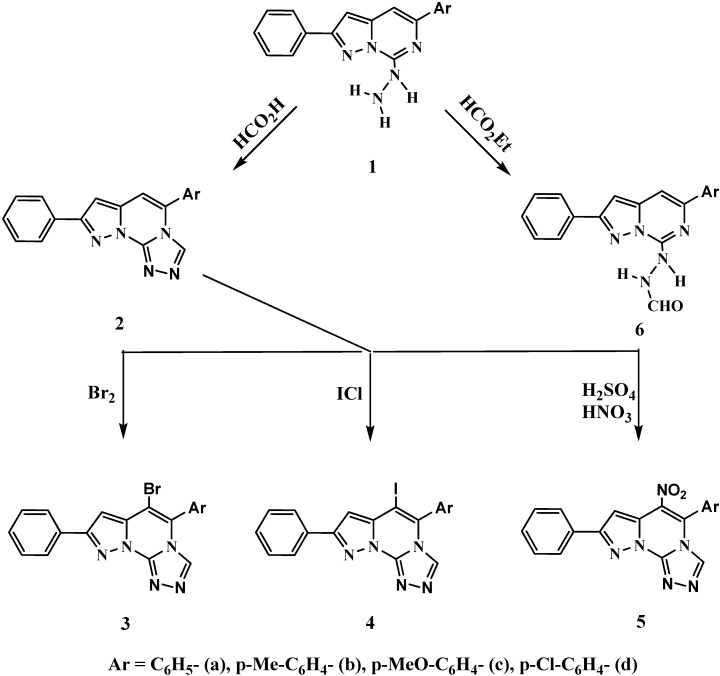
Synthesisandelectrophilicsubstitutionreactionsofpyrazolotriazolopyrimidines.

Much work from our laboratory has utilized hydrazino heterocycles as raw materials for the synthesis of various types of heterocyclic compounds [18,19,20,21]. In the present investigation, the target pyrazolotriazolopyrimidine compounds were synthesized from 5-aryl-7-hydrazino-2-phenyl-pyrazolo[1,5-*c*]pyrimidines **1a-d** that were prepared via a sequence of reactions from ethyl phenylpropiolate [2,22]. Heating of **1a-d** with formic acid under reflux yielded a novel series of 5-aryl-8-phenylpyrazolo[1,5-*c*]-1,2,4-triazolo[4,3-*a*]pyrimidines **2a-d** (Scheme 1). The structures of **2a-d** were deduced from their spectral analyses. Thus, the ^1^H-NMR spectra revealed the presence of three singlets for the pyrazole ring proton at δ_H_ 6.91–7.33 ppm, of the pyrimidine ring proton at δ_H_ 7.43–7.44 ppm and of triazole ring proton at δ_H_ 8.53–9.00 ppm, in addition to the aromatic ring protons and the absence of NH signals. The MS spectra also showed a molecular ion peak as a base peak that indicated the stability of this ring.

The electrophilic substitution reactions of pyrazolotriazolopyrimidines **2a-d** such as bromination with bromine, iodination with iodine monochloride and nitration with nitric and sulfuric acids in glacial acetic acid gave the respective 5-aryl-6-bromo-, 5-aryl-6-iodo- and 5-aryl-6-nitro-8- phenylpyrazolo[1,5-*c*]-1,2,4-triazolo[4,3-*a*]pyrimidines **3a-d**, **4a-d** and **5a-d**. Their ^1^H-NMR spectra revealed the absence of signals due to the pyrimidine ring proton and the presence of a pyrazole ring proton signal at δ_H_ 6.81–7.33 ppm and a triazole ring proton singlet at δ_H_ 8.52–9.04 ppm, together with the aromatic proton signals at δ_H_ 7.12–8.14 ppm. The structure of these derivatives were also confirmed from their mass spectral data.

Treatment of **1a-d** with boiling ethyl formate afforded 5-aryl-7-formylhydrazino-2- phenylpyrazolo[1,5-*c*]pyrimidines **6a-d**. Their ^1^H-NMR spectra showed a new characteristic signal at δ_H_ 7.98–8.08 ppm corresponding to the formyl proton, in addition to the aromatic ring protons at δ_H_ 7.20–7.99 ppm, with other characteristic signals; a singlet for the exchangeable two NH protons which were assigned at δ_H_ 4.73–4.80 ppm, a singlet at δ_H_ 6.68–6.72 ppm for the pyrazole ring proton and a singlet at δ_H_ 7.19–7.29 ppm for the pyrimidine ring proton.

**Scheme 2:**
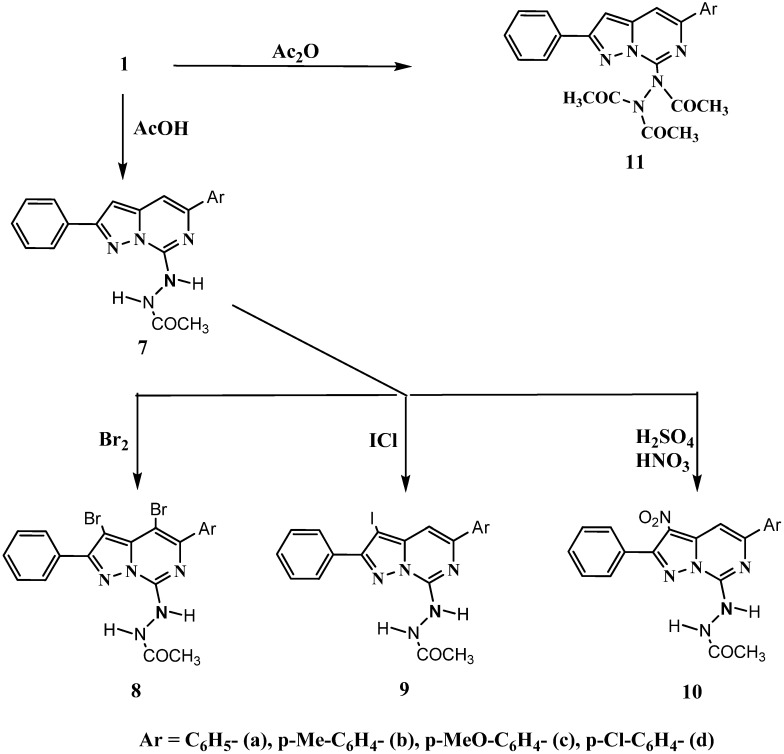
Synthesisandelectrophilicsubstitutionreactionsof7-acetyl-hydrazinopyrazolopyrimidines.

Boiling of hydrazine derivatives **1a-d** with acetic acid under reflux afforded 7-acetylhydrazino-5- aryl-2-phenylpyrazolo[1,5-*c*]pyrimidines **7a-d** (Scheme 2). The structures of **7a-d** were confirmed by their ^1^H-NMR spectra, which revealed an acetyl group proton singlet at δ_H_ 2.04–2.36 ppm, in addition to the characteristic signals of pyrazole and pyrimidine ring protons, two exchangeable NH protons and aromatic ring protons. The mass spectra of **7a-d** which showed their molecular ion peaks as a base peak also confirmed the structures.

Next, the electrophilic substitution reaction of **7a-d** via bromination with bromine in acetic acid gave the unexpected dibromo derivatives **8a-d** rather than the monobromo derivatives. Their ^1^H-NMR spectra showed the absence of the signals of both pyrazole and pyrimidine ring protons and the presence of acetyl group protons, in addition to the other characteristic signals. These unexpected obtained products may be due to the excess bromine added to obtain a homogenous reaction mixture.

Iodination and nitration of **7a-d** yielded the expected 5-aryl-2-phenyl-3-substituted-pyrazolo[1,5- *c*]pyrimidine derivatives **9a-d** and **10a-d**, respectively . Their ^1^H-NMR spectra revealed the absence of pyrazole ring proton and the presence of pyrimidine ring proton at δ_H_7.18–7.73 ppm as well as the other characteristic signals.

Furthermore, acetylation of **1a-d** with boiling acetic anhydride afforded the triacetyl derivatives **11a-d**. Their ^1^H-NMR spectra revealed the absence of the NH protons of the starting hydrazine derivatives and the presence of signals at δ_H_ 2.42–2.56 ppm corresponding to the three acetyl groups protons, as well as a singlet at δ_H_ 6.87–7.28 ppm for the pyrazole ring proton and a singlet at δ_H_ 7.70–8.24 ppm for the pyrimidine ring proton, in addition to the aromatic ring protons at δ_H_ 6.98–8.05 ppm. The structures of **11a-d** were also confirmed by their MS spectra which showed fragmentation process involving a sequential elimination of two ketene molecules to give the most stable one (M^.+^-2CH_2_CO) as a base peak.

## 3. Experimental

### 3.1. General

Melting points were determined on a Kofler Block and are uncorrected. Elemental analyses were carried out in the Microanalytical Laboratory of the Faculty of Science, Cairo University. The IR spectra of compounds were recorded on a Bruker Tensor 37 Fourier Transform infrared 8400 spectrophotometer using potassium bromide pellets and frequencies are reported in cm^−1^. The ^1^H- NMR spectra were recorded on a JEOL JNM ECA 500 MHZ instrument and chemical shifts δ_H_ are given in ppm relative to tetramethylsilane used as internal standard. Mass spectra were recorded at 70 ev with a GCMS-QP 1000 EX spectrometer. Reactions were routinely followed by thin layer chromatography (TLC; Merck Kieselgel60-F254 precoated plastic plates). The spots were detected by iodine. 5-Aryl-7-hydrazino-2- phenylpyrazolo[1,5-*c*]pyrimidines **1** were prepared from the respective acetylenic β-diketones as described earlier [2,22].

### 3.2. Synthesis of Compounds

#### 3.2.1. 5-Aryl-8-phenylpyrazolo[1,5-*c*]-1,2,4-triazolo[4,3-*a*]pyrimidines **2a-d**

A mixture of 5-aryl-7-hydrazino-2-phenylpyrazolo[1,5-*c*]pyrimidines (**1a-d**, 1 mmol) and formic acid (10 mL, 99%) was heated under reflux for 10 h. The mixture was evaporated under reduced pressure and the obtained residue was triturated with water, filtered, washed with EtOH and crystallized from EtOH to give the 5-aryl-8-phenylpyrazolo[1,5-*c*]-1,2,4-triazolo[4,3-*a*]pyrimidines **2a-d** as colorless needles.

*5,8-Diphenylpyrazolo[1,5-c]-1,2,4-triazolo[4,3-a]pyrimidine* (**2a**). Yield 81%, 0.25 g, mp 245–246 °C; IR (υ_max_, cm^−1^): 1649 (pyrazole ring C=N), 1580 (triazole ring C=N), and 1476 (C=C); ^1^H-NMR (CDCl_3_, δ_H_, ppm): 6.91 (s, 1H, pyrazole-H), 7.43 (s, 1H, pyrimidine-H), 7.45–7.64 (m, 8H, aromatic- H), 8.05 (d, 2H, aromatic-H) and 8.53 (s, 1H, triazole-H); MS, *m/z* (%) = 312 (M^.+^+1, 100,), 285 (M^.+^- CN, 4), 283 (M^.+^-N_2_, 33), 257 (M^.+^-CN_3_, 3), 255 (M^.+^-CH_2_N_3_, 16) and 227 (M^.+^-CH_2_N_5_, 8); Anal. Calc. for C_19_H_13_N_5_ (311.34): C, 73.30; H, 4.21; N, 22.49%, found: C, 73.27; H, 4.22; N, 22.53%. 

*8-Phenyl-5-p-tolylpyrazolo[1,5-c]-1,2,4-triazolo[4,3-a]pyrimidine* (**2b**). Yield 76%, 0.25 g, mp 247–248 °C; IR (υ_max_, cm^−1^): 1643 (pyrazole ring C=N), 1577 (triazole ring C=N), and 1454 (C=C); ^1^H NMR (DMSO-*d6*, δ_H_, ppm): 2.39 (s, 3H, CH3), 7.03 (d, 2H, aromatic-H), 7.26 (s, 1H, pyrazole-H),7.39–7.51 (m, 5H, aromatic-H), 7.44 (s, 1H, pyrimidine-H), 7.65 (d, 2H, aromatic-H) and 8.96 (s, 1H, triazole-H); MS, *m/z* (%) = 327 (M^.+^+2, 17), 325 (M^.+^, 100), 297 (M^.+^-N2, 24), 282 (M^.+^-CH_3_N_2_, 8),255 (M.+-C_2_H_4_N_3_, 7) and 227 (M^.+^-C_2_H_4_N_5_, 10); Anal. Calc. for C_20_H_15_N_5_ (325.37): C, 73.83; H, 4.65;N, 21.52%, found: C, 73.87; H, 4.62; N, 21.55%.

*5-(p-Methoxyphenyl)-8-phenylpyrazolo[1,5-c]-1,2,4-triazolo[4,3-a]pyrimidine* (**2c**). Yield 74%, 0.25 g, mp 237–238 °C; IR (υ_max_, cm^−1^): 1643 (pyrazole ring C=N), 1587 (triazole ring C=N), and 1454 (C=C); ^1^H NMR (CDCl3, δ_H_, ppm): 3.92 (s, 3H, CH_3_), 6.93 (s, 1H, pyrazole-H), 7.10 (d, 2H, aromatic- H), 7.40–7.48 (m, 3H, aromatic-H), 7.44 (s, 1H, pyrimidine-H), 7.57 (d, 2H, aromatic-H), 8.05 (d, 2H, aromatic-H) and 8.55 (s, 1H, triazole-H); MS, *m/z* (%) = 343 (M^.+^+2, 15), 341 (M^.+^, 100), 326 (M^.+^- CH_3_, 3), 313(M^.+^-N_2_, 10), 299 (M^.+^-CH_2_N_2_, 8), 285 (M^.+^-C_2_H_4_N_2_, 2) and 270 (M^.+^-C_2_H_3_N_2_O, 11); Anal. Calc. for C_20_H_15_N_5_O (341.37): C, 70.37; H, 4.43; N, 20.52%, found: C, 70.40; H, 4.40; N, 20.55%.

*5-(p-Chlorophenyl)-8-phenylpyrazolo[1,5-c]-1,2,4-triazolo[4,3-a]pyrimidine* (**2d**). Yield 71%, 0.25 g, mp 299–300 °C; IR (υ_max_, cm^−1^): 1643 (pyrazole ring C=N), 1583 (triazole ring C=N), and 1467 (C=C); ^1^H NMR (DMSO-*d_6_*, δ_H_, ppm): 7.33 (s, 1H, pyrazole-H), 7.43 (s, 1H, pyrimidine-H), 7.44–7.52 (m, 3H, aromatic-H), 7.66 (d, 2H, aromatic-H), 7.79 (d, 2H, aromatic-H), 8.04 (d, 2H, aromatic-H) and 9.00 (s, 1H, triazole-H); MS, *m/z* (%) = 347 (M^.+^+1, 52), 345 (M^.+^-1, 100), 319 (M^.+^-HCN, 8), 317 (M^.+^-HN_2_, 17), 289 (M^.+^-C_2_H_5_N_2_, 6), 282 (M^.+^-HClN_2_, 13), 255 (M^.+^-C_2_H_4_ClN_2_, 16) and 227 (M^.+^- C_3_H_6_ClN_3_, 8); Anal. Calc. for C_19_H_12_ClN_5_ (345.79): C, 66.00; H, 3.50; N, 20.25%, found: C, 59.98; H, 3.50; N, 20.22%.

#### 3.2.2. 5-Aryl-6-bromo-8-phenylpyrazolo[1,5-*c*]-1,2,4-triazolo[4,3-*a*]pyrimidines **3a-d**

A solution of bromine (0.06 mL, 1.2 mmol) in acetic acid (10 mL) was gradually added to a suspension of 5-aryl-8-phenylpyrazolo[1,5-*c*]-1,2,4-triazolo[4,3-*a*]pyrimidines **2a-d** (1 mmol) in acetic acid (10 mL) with stirring for 3 h at room temperature. The precipitated 5-aryl-6-bromo-8- phenylpyrazolo[1,5-*c*]-1,2,4-triazolo-[4,3-*a*]pyrimidines **3a-d** were filtered, washed with water, dried and crystallized from EtOH as colorless needles.

*6-Bromo-5,8-diphenylpyrazolo[1,5-c]-1,2,4-triazolo[4,3-a]pyrimidine* (**3a**). Yield 75%, 0.30 g, mp 235–236 °C; IR (υ_max_, cm^−1^): 1640 (pyrazole ring C=N), 1580 (triazole ring C=N), and 1455 (C=C); ^1^H NMR (CDCl_3_, δ_H_, ppm): 6.92 (s, 1H, pyrazole-H), 7.43–7.69 (m, 8H, aromatic-H), 8.14 (d, 2H, aromatic-H) and 8.58 (s, 1H, triazole-H); MS, *m/z* (%) = 390 (M^.+^, 100), 362 (M^.+^-N_2_, 13), 310 (M^.+^- Br, 2), 282 (M^.+^-BrN_2_, 13), 255 (M^.+^-CHBrN_3_, 12) and 227 (M^.+^-CHBrN_5_, 6); Anal. Calc. for C_19_H_12_BrN_5_ (390.24): C, 58.48; H, 3.10; N, 17.95%, found: C, 58.52; H, 3.08; N, 17.90%.

*6-Bromo-8-phenyl-5-p-tolylpyrazolo[1,5-c]-1,2,4-triazolo[4,3-a]pyrimidine* (**3b**). Yield 75%, 0.30 g, mp 215–216 °C; IR (υ_max_, cm^−1^): 1636 (pyrazole ring C=N), 1578 (triazole ring C=N), and 1421 (C=C); ^1^H NMR (CDCl_3_, δ_H_, ppm): 2.49 (s, 3H, CH_3_), 6.90 (s, 1H, pyrazole-H), 7.41–7.57 (m, 7H, aromatic-H), 8.14 (d, 2H, aromatic-H) and 8.60 (s, 1H, triazole-H); MS, *m/z* (%) = 407 (M^.+^+3, 8), 405 (M^.+^+1, 100), 377 (M^.+^-HCN, 8), 324 (M^.+^-Br, 2), 281 (M^.+^-CH_3_BrN_2_, 6) and 254 (M^.+^-C_2_H_4_BrN_3_, 5); Anal. Calc. for C_20_H_14_BrN_5_ (404.26): C, 59.42; H, 3.49; N, 17.32%, found: C, 59.39; H, 3.45; N, 17.27%.

*6-Bromo-5-(p-methoxyphenyl)-8-phenylpyrazolo[1,5-c]-1,2,4-triazolo[4,3-a]pyrimidine* (**3c**). Yield 71%, 0.30 g, mp 213–214 °C; IR (υ_max_, cm^−1^): 1632 (pyrazole ring C=N), 1587 (triazole ring C=N), and 1427 (C=C); ^1^H NMR (CDCl_3_, δ_H_, ppm): 3.90 (s, 3H, OCH_3_), 6.81 (s, 1H, pyrazole-H),7.09 (d, 2H, aromatic-H), 7.44–7.56 (m, 3H, aromatic-H),7.59 (d, 2H, aromatic-H), 8.10 (d, 2H, aromatic-H) and 8.57 (s, 1H, triazole-H); MS, *m/z* (%) = 422 (M^.+^+2, 100), 420 (M^.+^, 80), 405 (M^.+^-CH_3_, 3), 391 (M^.+^- HN_2_, 8), 378 (M^.+^-CH_2_N_2_, 5), 350 (M^.+^- C_2_H_4_N_3_, 6), 340 (M^.+^-Br, 3), 313 (M^.+^-CHBrN, 4), 281 (M^.+^- CH_3_BrN_2_O, 5) and 269 (M^.+^-C_2_H_3_BrN_2_O, 13); Anal. Calc. for C_20_H_14_BrN_5_O (420.26): C, 57.16; H, 3.36; N, 16.66%, found: C, 57.20; H, 3.38; N, 16.70%.

*6-Bromo-5-(p-chlorophenyl)-8-phenylpyrazolo[1,5-c]-1,2,4-triazolo[4,3-a]pyrimidine* (**3d**). Yield 71%, 0.30 g, mp 238–239 °C; IR (υ_max_, cm^−1^): 1637 (pyrazole ring C=N), 1580 (triazole ring C=N), and 1414 (C=C); ^1^H NMR (CDCl_3_, δ_H_, ppm): 6.92 (s, 1H, pyrazole-H), 7.47–7.66 (m, 7H, aromatic-H), 8.14 (d, 2H, aromatic-H) and 8.55 (s, 1H, triazole-H); MS, *m/z* (%) = 429 (M^.+^+4, 3), 428 (M^.+^+3, 38), 426 (M^.+^+1, 100), 424 (M^.+^-1, 76), 398 (M^.+^-HCN, 11), 317 (M^.+^-BrN_2_, 8), 289 (M^.+^-CH_2_BrN_3_, 8), 281 (M^.+^- HBrClN_2_, 15), 253 (M^.+^-C_2_H_5_BrClN_2_, 10) and 226 (M^.+^-C_3_H_6_BrClN_3_, 7); Anal. Calc. for C_19_H_11_BrClN_5_ (424.68): C, 53.74; H, 2.61; N, 16.49%; found: C, 53.72; H, 2.57; N, 16.50%.

#### 3.2.3. 5-Aryl-6-iodo-8-phenylpyrazolo[1,5-*c*]-1,2,4-triazolo[4,3-*a*]pyrimidines **4a-d**

A solution of iodine monochloride (0.2 g, 1.2 mmol) in acetic acid (10 mL) was gradually added to a suspension of 5- aryl-8-phenylpyrazolo[1,5-*c*]-1,2,4-triazolo[3,4-*a*]pyrimidines **2a-d** (1mmol) in acetic acid (10 mL) with stirring for 3 h at room temperature. The reaction mixture was then poured onto crushed ice and the precipitated 5-aryl-6-iodo-8-phenylpyrazolo[1,5,*c*]-1,2,4-triazolo[3,4- *a*]pyrimidines **4a-d** were filtered, washed with water, dried and crystallized from EtOH as colorless needles.

*6-Iodo-5,8-diphenylpyrazolo[1,5-c]-1,2,4-triazolo[4,3-a]pyrimidine* (**4a**). Yield 91%, 0.40 g, mp 281–282 °C; IR (υ_max_, cm^-1^): 1640 (pyrazole ring C=N), 1575 (triazole ring C=N), and 1405 (C=C); ^1^H NMR (CDCl_3_, δ_H_, ppm): 6.94 (s, 1H, pyrazole-H), 7.50-7.69 (m, 8H, aromatic-H), 8.10 (d, 2H, aromatic-H) and 8.61 (s, 1H, triazole-H); MS, *m/z* (%) = 437 (M^.+^, 100), 409 (M^.+^-N_2_, 5), 310 (M^.+^-I,7), 282 (M^.+^-IN_2_, 10), 242 (M^.+^-C_2_H_2_IN_3_, 4) and 227 (M^.+^-C_3_H_5_IN_3_, 8); Anal. Calc. for C_19_H_12_IN_5_ (437.24): C, 52.19; H, 2.77; N, 16.02%, found: C, 52.17; H, 2.80; N, 16.00%.

*6-Iodo-8-phenyl-5-p-tolylpyrazolo[1,5-c]-1,2,4-triazolo[4,3-a]pyrimidine* (**4b**). Yield 89%, 0.40 g, mp 245–246 °C; IR (υ_max_, cm^−1^): 1633 (pyrazole ring C=N), 1576 (triazole ring C=N), and 1416 (C=C); ^1^H NMR (CDCl_3_, δ_H_, ppm): 2.49 (s, 3H, CH_3_), 6.90 (s, 1H, pyrazole-H), 7.41-7.69 (m, 7H, aromatic-H), 8.08 (d, 2H, aromatic-H) and 8.60 (s, 1H, triazole-H); MS, *m/z* (%) = 453 (M^.+^+2, 9), 452 (M^.+^+1, 100), 423 (M^.+^-N_2_, 5), 325 (M^.+^+1-I, 12), 296 (M^.+^-IN_2_, 9) and 269 (M^.+^-CHIN_3_, 10); Anal. Calc. for C_20_H_14_IN_5_ (451.26): C, 53.23; H, 3.13; N, 15.52%, found: C, 53.27; H, 3.15; N, 15.50%.

*6-Iodo-5-(p-methoxyphenyl)-8-phenylpyrazolo[1,5-c]-1,2,4-triazolo[4,3-a]pyrimidine* (**4c**). Yield 85%, 0.40 g, mp 225–226 °C; IR (υ_max_, cm^−1^): 1623 (pyrazole ring C=N), 1572 (triazole ring C=N), and 1458 (C=C); ^1^H NMR (CDCl_3_, δ_H_, ppm): 3.93 (s, 3H, OCH_3_), 6.86 (s, 1H, pyrazole-H), 7.12 (d, 2H, aromatic-H), 7.48–7.51 (m, 3H, aromatic-H), 7.61 (d, 2H, aromatic-H), 8.10 (d, 2H, aromatic-H) and 8.61 (s, 1H, triazole-H); MS, *m/z* (%) = 470 (M^.+^+3, 3), 468 (M^.+^+1, 100),439 (M^.+^-N_2_, 6), 341 (M^.+^+1-I, 12), 313 (M^.+^-CHIN, 4), 285 (M^.+^-CHIN_3_, 7), 269 (M^.+^-C_2_H_3_IN_2_O, 12) and 255 (M^.+^- C_2_H_3_IN_3_O, 4); Anal. Calc. for C_20_H_14_IN_5_O (467.26): C, 51.41; H, 3.02; N, 14.99%, found: C, 51.44; H, 3.00; N, 15.02%.

*5-(p-Chlorophenyl)-6-iodo-8-phenylpyrazolo[1,5-c]-1,2,4-triazolo[4,3-a]pyrimidine* (**4d**). Yield 85%, 0.40 g, mp 270–271 °C; IR (υ_max_, cm^−1^): 1632 (pyrazole ring C=N), 1576 (triazole ring C=N), and 1410 (C=C); ^1^H NMR (DMSO-*d_6_*, δ_H_, ppm): 7.02 (s, 1H, pyrazole-H), 7.49–7.54 (m, 3H, aromatic-H), 7.65 (d, 2H, aromatic-H), 7.80 (d, 2H, aromatic-H), 7.92 (d, 2H, aromatic-H) and 9.04 (s, 1H, triazole-H); MS, *m/z* (%) = 475 (M^.+^+3, 3), 472 (M^.+^, 100), 443 (M^.+^-HN_2_, 3), 345 (M^.+^-I, 7), 309 (M^.+^-ClI, 2), 289 (M^.+^-CH_2_IN_3_, 5), 282 (M^.+^-CHClIN, 8) and 241 (M^.+^-C_2_H_2_ClIN_3_, 9); Anal. Calc. for C_19_H_11_ClIN_5_ (471.68): C, 48.38; H, 2.35; N, 14.85%, found: C, 48.40; H, 2.40; N, 14.90%.

#### 3.2.4. 5-Aryl-6-nitro-8-phenylpyrazolo[1,5-*c*]-1,2,4-triazolo[4,3-*a*]pyrimidines **5a-d**

A mixture of nitric acid (d 1.41, 1 mL) and sulfuric acid (d 1.84, 1 mL) in glacial acetic acid (10 mL) was gradually added to a suspension of 5-aryl-8-phenylpyrazolo[1,5-*c*]-1,2,4-triazolo[4,3- *a*]pyrimidines **2a-d** (1 mmol) in glacial acetic acid (10 mL) with stirring for 3 h at room temperature. The reaction mixture was then poured onto cold water with stirring and the yellow precipitated solids were filtered, washed with cold water, dried and crystallized from EtOH to give the title compounds **5a-d** as yellow needles.

*6-Nitro-5,8-diphenylpyrazolo[1,5-c]-1,2,4-triazolo[4,3-a]pyrimidine* (**5a**). Yield 83%, 0.30 g, mp 241–242 °C; IR (υ_max_, cm^−1^): 1649 (pyrazole ring C=N), 1579 (triazole ring C=N), and 1462 (C=C); ^1^H NMR (CDCl_3_, δ_H_, ppm): 6.93 (s, 1H, pyrazole-H), 7.40–7.44 (m, 6H, aromatic-H), 7.62 (d, 2H, aromatic-H), 8.03 (d, 2H, aromatic-H) and 8.52 (s, 1H, triazole-H); MS, *m/z* (%) = 356 (M^.+^, 7), 326 (M^.+^-H_2_N_2_, 4), 311 (M^.+^+1-NO_2_, 100), 283 (M^.+^-CHN_2_O_2_, 27), 271 (M^.+^ +1-CN_3_O_2_, 2) and 255 (M^.+^-CHN_4_O_2_, 13); Anal. Calc. for C_19_H_12_N_6_O_2_ (356.34): C, 64.04; H, 3.39; N, 23.58%, found: C, 64.00; H, 3.40; N, 23.60%.

*6-Nitro-8-phenyl-5-p-tolylpyrazolo[1,5-c]-1,2,4-triazolo[4,3-a]pyrimidine* (**5b**). Yield 81%, 0.30 g, mp 243–244 °C; IR (υ_max_, cm^−1^): 1643 (pyrazole ring C=N), 1578 (triazole ring C=N), and 1415 (C=C); ^1^H NMR (CDCl_3_, δ_H_, ppm): 2.47 (s, 3H, CH_3_), 6.90 (s, 1H, pyrazole-H), 7.37–7.45 (m, 5H, aromatic-H), 7.51 (d, 2H, aromatic-H), 8.03 (d, 2H, aromatic-H) and 8.54 (s, 1H, triazole-H); MS, *m/z* (%) = 370 (M^.+^, 4), 340 (M^.+^-H_2_N_2_, 2), 325 (M^.+^+1-NO_2_, 100), 309 (M^.+^-CH_3_NO_2_, 1), 297 (M^.+^-CHN_2_O_2_, 22) and 269 (M^.+^-C_2_H_3_N_3_O_2_, 9); Anal. Calc. for C_20_H_14_N_6_O_2_ (370.36): C, 64.86; H, 3.81; N, 22.69%, found: C, 64.90; H, 3.80; N, 22.72%.

*5-(p-Methoxyphenyl)-6-nitro-8-phenylpyrazolo[1,5-c]-1,2,4-triazolo[4,3-a]pyrimidine* (**5c**), Yield 77%, 0.30 g, mp 250–251 °C; IR (υ_max_, cm^−1^): 1637 (pyrazole ring C=N), 1585 (triazole ring C=N), and 1420 (C=C); ^1^H NMR (CDCl_3_, δ_H_, ppm): 3.89 (s, 3H, OCH_3_), 6.97 (s, 1H, pyrazole-H), 7.45–7.47 (m, 3H, aromatic-H), 7.62 (d, 2H, aromatic-H), 7.79 (d, 2H, aromatic-H), 8.02 (d, 2H, aromatic-H) and 8.75 (s, 1H, triazole-H); MS, *m/z* (%) = 388 (M^.+^+2, 15), 387 (M^.+^+1, 100), 356 (M^.+^-CH_2_O, 7), 339 (M^.+^-HNO_2_, 7), 312 (M^.+^-CH_2_N_2_O_2_, 4), 310 (M^.+^-CH_4_N_2_O_2_, 11), 284 (M^.+^-C_3_H_6_N_2_O_2_, 5) and 251 (M^.+^-C_7_H_7_N_2_O, 16); Anal. Calc. for C_20_H_14_N_6_O_3_ (386.36): C, 62.17; H, 3.65; N, 21.75%, found: C, 62.20; H, 3.61; N, 21.73%.

*5-(p-Chlorophenyl)-6-nitro-8-phenylpyrazolo[1,5-c]-1,2,4-triazolo[4,3-a]pyrimidine* (**5d**). Yield 77%, 0.30 g, mp 306–307 °C; IR (υ_max_, cm^−1^): 1641 (pyrazole ring C=N), 1583 (triazole ring C=N), and 1416 (C=C); ^1^H NMR (DMSO-*d_6_*, δ_H_, ppm): 7.33 (s, 1H, pyrazole-H), 7.43 (t, 1H, aromatic-H), 7.50 (t, 2H, aromatic-H), 7.66 (d, 2H, aromatic-H), 7.79 (d, 2H, aromatic-H), 8.03 (d, 2H, aromatic-H) and 8.99 (s, 1H, triazole-H); MS, *m/z* (%) = 391 (M^.+^, 3), 349 (M^.+^-CH_2_N_2_, 3), 347 (M^.+^-N_2_O, 46), 345 (M^.+^-NO_2_, 100), 317 (M^.+^-N_3_O_2_, 17), 289 (M^.+^-C_2_H_4_N_3_O_2_, 7), 282 (M^.+^-C_6_H_5_O_2_, 15), 254 (M^.+^-C_7_H_4_ClN, 22) and 227 (M^.+^-C_7_H_3_ClN_3_, 12); Anal. Calc. for C_19_H_11_ClN_6_O_2_ (390.78): C, 58.40; H, 2.84; N, 21.51%, found: C, 58.44; H, 2.80; N, 21.53%.

#### 3.2.5. 5-Aryl-7-formylhydrazino-2-phenylpyrazolo[1,5-*c*]pyrimidines **6a-d**

A suspension of 5-aryl-7-hydrazino-2-phenylpyrazolo[1,5-*c*]pyrimidines (**1a-d**, 1mmol) and ethyl formate (5 mL) was heated under reflux for 3 h. The product which separated upon cooling was filtered, washed with EtOH and crystallized from EtOH to give the title compounds **6a-d** as colorless needles.

*7-Formylhydrazino-2,5-Diphenylpyrazolo[1,5-c]pyrimidine* (**6a**). Yield 76%, 0.25 g, mp 177–178 °C; IR (_υmax_, cm^−1^): 3368 (NH), 1700 (C=O), 1624 (pyrazole ring C=N), 1568 (pyrimidine ring C=N), and 1455 (C=C); ^1^H NMR (CDCl_3_, δ_H_, ppm): 4.80 (s, 2H, exchangeable 2NH), 6.72 (s, 1H, pyrazole-H), 7.29 (s, 1H, pyrimidine-H), 7.30-7.49 (m, 8H, aromatic-H), 7.98 (d, 2H, aromatic-H) and 8.08 (s, 1H, CHO); MS, *m/z* (%) = 330 (M^.+^+1, 2), 302 (M^.+^+1-CO, 100), 286 (M^.+^-CHNO, 23), 272 (M^.+^-CHN_2_O, 81), 257 (M^.+^-C_2_H_4_N_2_O, 2), 244 (M^.+^-C_2_H_3_N_3_O, 17) and 228 (M^.+^-C_2_H_5_N_4_O, 6); Anal. Calc. for C_19_H_15_N_5_O (329.36): C, 69.29; H, 4.59; N, 21.26%, found: C, 69.30; H, 4.62; N, 21.30%.

*7-Formylhydrazino-2-phenyl-5-p-tolylpyrazolo[1,5-c]pyrimidine* (**6b**). Yield 71%, 0.25 g, mp 132–133 °C; IR (υ_max_, cm^−1^): 3315 (NH), 1701 (C=O), 1610 (pyrazole ring C=N), 1572 (pyrimidine ring C=N), and 1447 (C=C); ^1^H NMR (CDCl_3_, δ_H_, ppm): 2.42 (s, 3H, CH_3_), 4.77 (s, 2H, exchangeable 2NH), 6.70 (s, 1H, pyrazole-H), 7.28 (s, 1H, pyrimidine-H), 7.29-7.49 (m, 7H, aromatic-H), 7.96 (d, 2H, aromatic- H) and 7.98 (s, 1H, CHO); MS, *m/z* (%) = 344 (M^.+^+1, 2), 316 (M^.+^+1-CO, 100), 300 (M^.+^-CHNO, 33), 286 (M^.+^-CHN_2_O, 85), 270 (M^.+^-C_2_H_5_N_2_O, 4), 259 (M^.+^-C_2_H_2_N_3_O, 7) and 227 (M^.+^-C_8_H_6_N, 10); Anal. Calc. for C_20_H_17_N_5_O (343.38): C, 69.96; H, 4.99; N, 20.40%, found: C, 70.02; H, 5.02; N, 20.44%

*7-Formylhydrazino-5-(p-methoxyphenyl)-2-phenylpyrazolo[1,5-c]pyrimidine* (**6c**). Yield 69%, 0.25 g, mp 157–158 °C; IR (υ_max_, cm^−1^): 3265(NH), 1708 (C=O), 1667 (pyrazole ring C=N), 1585 (pyrimidine ring C=N), and 1448 (C=C); ^1^H NMR (CDCl_3_, δ_H_, ppm): 3.87 (s, 3H, OCH_3_), 4.73 (s, 2H, exchangeable 2NH), 6.68 (s, 1H, pyrazole-H), 7.19 (s, 1H, pyrimidine-H), 7.20–7.46 (m, 3H, aromatic- H), 7.95–7.97 (m, 4H, aromatic-H), 7.98 (d, 2H, aromatic-H) and 8.00 (s, 1H, CHO); MS, *m/z* (%) = 361 (M^.+^+2, 5), 359 (M^.+^, 30), 332 (M^.+^+1-CO, 100), 316 (M^.+^-CHNO, 23), 302 (M^.+^-CHN_2_O, 78), 287 (M^.+^-C_2_H_4_N_2_O, 12) and 275 (8, M^.+^-C_2_H_2_N_3_O); Anal. Calc. for C_20_H_17_N_5_O_2_ (359.38): C, 66.84; H, 4.77; N, 19.49%, found: C, 66.80; H, 4.82; N, 19.50%.

*5-(p-Chlorophenyl)-7-formylhydrazino-2-phenylpyrazolo[1,5-c]pyrimidine* (**6d**). Yield 69%, 0.25 g, mp 175–176 °C; IR (υ_max_, cm^−1^): 3315 (NH), 1700 (C=O), 1615 (pyrazole ring C=N), 1575 (pyrimidine ring C=N), and 1450 (C=C); ^1^H NMR (CDCl_3_, δ_H_, ppm): 4.76 (s, 2H, exchangeable 2NH), 6.72 (s, 1H, pyrazole-H), 7.28 (s, 1H, pyrimidine-H), 7.41–7.46 (m, 5H, aromatic-H), 7.95–7.99 (m, 4H, aromatic- H) and 8.00 (s, 1H, CHO); MS, *m/z* (%) = 361 (M^.+^-3, 2,), 335 (M^.+^+1-CO, 19), 320 (M^.+^-CHNO, 100), 305 (M^.+^-CH_2_N_2_O, 47), 294 (M^.+^-C_2_HN_2_O, 10), 269 (M^.+^-C_6_H_6_O, 5) and 242 (M^.+^-C_7_H_7_NO, 5); Anal. Calc. for C_19_H_14_ClN_5_O (363.80): C, 62.73; H, 3.88; N, 19.25%, found: C, 62.70; H, 3.90; N, 19.30%.

#### 3.2.6. 7-Acetylhydrazino-5-aryl-2-phenylpyrazolo[1,5-*c*]pyrimidines **7a-d**

5-Aryl-7-hydrazino-2-phenylpyrazolo[1,5-*c*]pyrimidines **1a-d** (1mmol) and glacial acetic acid (10 mL) was heated at reflux for 3 h. The reaction mixture was poured onto crushed ice and the product which separated was filtered, washed with water, dried and crystallized from EtOH to give the title compounds **7a-d** as colorless needles.

*7-Acetylhydrazino-2,5-diphenylpyrazolo[1,5-c]pyrimidine* (**7a**). Yield 88%, 0.30 g, mp 217–218 °C; IR (υ_max_, cm^−1^): 3150 (NH), 1661 (C=O), 1628 (pyrazole ring C=N), 1568 (pyrimidine ring C=N), and 1459 (C=C); ^1^H NMR (CDCl_3_, δ_H_, ppm): 2.21(s, 3H, COCH_3_), 6.67 (s, 1H, pyrazole-H), 7.21 (s, 1H, pyrimidine-H), 7.30–7.51 (m, 5H, aromatic-H), 7.84–7.96 (m, 5H, aromatic-H), 8.01 (s, 1H, exchangeable NH) and 8.48 (s, 1H, exchangeable NHCO); MS, *m/z* (%) = 343 (M^.+^, 100), 328 (M^.+^-CH_3_, 1), 301 (M^.+^-COCH_2_, 59), 286 (M^.+^-NCOCH_3_, 18) and 243 (M^.+^-C_3_H_6_N_3_O, 11); Anal. Calc. for C_20_H_17_N_5_O (343.38): C, 69.96; H, 4.99; N, 20.40%, found: C, 70.00; H, 5.02; N, 20.43%.

*7-Acetylhydrazino-2-phenyl-5-p-tolylpyrazolo[1,5-c]pyrimidine* (**7b**). Yield 83%, 0.30 g, mp 224–225 °C; IR (υ_max_, cm^−1^): 3252 (NH), 1659 (C=O), 1618 (pyrazole ring C=N), 1556 (pyrimidine ring C=N), and 1445 (C=C); ^1^H NMR (CDCl_3_, δ_H_, ppm): 2.23(s, 3H, CH_3_), 2.36 (s, 3H, COCH_3_), 6.70 (s, 1H, pyrazole-H), 7.19 (d, 2H, aromatic-H), 7.26 (s, 1H, pyrimidine-H), 7.36–7.48 (m, 3H, aromatic-H), 7.79 (d, 2H, aromatic-H), 7.97 (d, 2H, aromatic-H), 8.14 (s, 1H, exchangeable NH) and 8.31 (s, 1H, exchangeable NHCO); MS, *m/z* (%) = 358 (M^.+^+1, 100), 315 (M^.+^-COCH_2_, 63), 300 (M^.+^-NCOCH_3_, 19), 288 (M^.+^-C_3_H_3_NO, 6) and 259 (M^.+^-C_3_H_4_N_3_O, 5); Anal. Calc. for C_21_H_19_N_5_O (357.41): C, 70.57; H, 5.36; N, 19.59%, found: C, 70.60; H, 5.32; N, 19.55%.

*7-Acetylhydrazino-5-p-methoxyphenyl-2-phenylpyrazolo[1,5-c]pyrimidine* (**7c**). Yield 81%, 0.30 g, mp 193–194 °C; IR (υ_max_, cm^−1^): 3283 (NH), 1664 (C=O), 1618 (pyrazole ring C=N), 1564 (pyrimidine ring C=N), and 1448 (C=C); ^1^H NMR (CDCl_3_, δ_H_, ppm): 2.24 (s, 3H, COCH_3_), 3.78 (s, 3H, OCH_3_), 6.63 (s, 1H, pyrazole-H), 6.88 (d, 2H, aromatic-H), 7.07 (s, 1H, pyrimidine-H), 7.36–7.50 (m, 3H, aromatic-H), 7.76 (d, 2H, aromatic-H), 7.91 (d, 2H, aromatic-H), 8.13 (s, 1H, exchangeable NH) and 8.91 (s, 1H, exchangeable NHCO); MS, *m/z* (%) = 374 (M^.+^+1, 100), 331 (M^.+^-COCH_2_, 38), 316 (M^.+^-NCOCH_3_, 16), 302 (M^.+^-NNCOCH_3_, 56), 287 (M^.+^-C_3_H_6_N_2_O, 8) and 259 (M^.+^- C_4_H_8_N_3_O, 19); Anal. Calc. for C_21_H_19_N_5_O_2_ (373.41): C, 67.55; H, 5.13; N, 18.76%, found: C, 67.58; H, 5.16; N, 18.80%.

*7-Acetylhydrazino-5-p-Chlorophenyl-2-phenylpyrazolo[1,5-c]pyrimidine* (**7d**). Yield 79%, 0.30 g, mp 246–247 °C; IR (υ_max_, cm^−1^): 3306 (NH), 1664 (C=O), 1613 (pyrazole ring C=N), 1572 (pyrimidine ring C=N), and 1447 (C=C); ^1^H NMR (DMSO-*d_6_*, δ_H_, ppm): 2.04 (s, 3H, COCH_3_), 7.08 (s, 1H, pyrazole-H), 7.67 (s, 1H, pyrimidine-H), 7.41–7.52 (m, 5H, aromatic-H), 8.09 (d, 2H, aromatic-H), 9.81 (s, 1H, exchangeable NH) and 10.14 (s, 1H, exchangeable NHCO); MS, *m/z* (%) = 382 (M^.+^+4, 1), 381 (M^.+^+3, 4), 379 (M^.+^+1, 44), 378 (M^.+^, 100), 336 (M^.+^-COCH_2_, 65), 320 (M^.+^-NHCOCH_3_, 16), 306 (M^.+^-NNHCOCH_3_, 77) and 270 (M^.+^-C_2_H_5_ClN_2_O, 10); Anal. Calc. for C_20_H_16_ClN_5_O (377.83): C, 63.58; H, 4.27; N, 18.54%, found: C, 63.61; H, 4.26; N, 18.60%.

#### 3.2.7. 7-Acetylhydrazino-5-aryl-3,4-dibromo-2--phenylpyrazolo[1,5-*c*]pyrimidines **8a-d**

A solution of bromine (0.12 mL, 2.4 mmol) in acetic acid (10 mL) was gradually added to a suspension of 7-acetylhydrazino-5-aryl-2-phenylpyrazolo[1,5-*c*]pyrimidines **7a-d** (1 mmol) in acetic acid (10 mL) with stirring for 3 h at room temperature. The precipitated 7-acetylhydrazino-5-aryl-3,4- dibromo-2-phenylpyrazolo[1,5-*c*]pyrimidines **8a-d** were filtered, washed with water, dried and crystallized from EtOH as colorless needles.

*7-Acetylhydrazino-3,4-dibromo-2,5-diphenylpyrazolo[1,5-c]pyrimidine* (**8a**). Yield 80%, 0.40 g, mp 229–230 °C; IR (υ_max_, cm^−1^): 3451 (NH), 1657 (C=O), 1644 (pyrazole ring C=N), 1560 (pyrimidine ring C=N), and 1439 (C=C); ^1^H NMR (CDCl_3_, δ_H_, ppm): 2.09 (s, 3H, COCH_3_), 7.44–7.53 (m, 6H, aromatic-H), 7.63 (d, 2H, aromatic-H), 7.94 (d, 2H, aromatic-H) and 8.01 (s, 2H, exchangeable NH and exchangeable NHCO); MS, *m/z* (%) = 505(M^.+^+4, 4), 503 (M^.+^+2, 38), 501 (M^.+^, 100), 459 (M^.+^- COCH_2_, 80), 444 (M^.+^-NCOCH_3_, 14), 429 (M^.+^-NNHCOCH_3_, 40), 423 (M^.+^+2-Br, 21), 421 (M^.+^-Br, 19) and 341 (M^.+^-Br2, 2); Anal. Calc. for C_20_H_15_Br_2_N_5_O (501.17): C, 47.93; H, 3.02; N, 13.97%, found: C, 47.95; H, 3.06; N, 14.00%.

*7-Acetylhydrazino-3,4-dibromo-2-phenyl-5-p-tolylpyrazolo[1,5-c]pyrimidine* (**8b**). Yield 77%, 0.40 g, mp 212–213 °C; IR (υ_max_, cm^−1^): 3280 (NH), 1664 (C=O), 1618 (pyrazole ring C=N), 1562 (pyrimidine ring C=N), and 1439 (C=C); ^1^H NMR (CDCl_3_, δ_H_, ppm): 2.10 (s, 3H, CH_3_), 2.40 (s, 3H, COCH_3_), 7.46–7.55 (m, 5H, aromatic-H), 7.86 (d, 2H, aromatic-H), 7.93 (d, 2H, aromatic-H), 8.04 (s, 1H, exchangeable NH) and 8.06 (s, 1H, exchangeable NHCO); MS, *m/z* (%) = 519 (M^.+^+4, 1), 517 (M^.+^+2, 15), 515 (M^.+^, 33), 473 (M^.+^-COCH_2_, 18), 458 (M^.+^-NCOCH_3_, 2), 444 (M^.+^-NNCOCH_3_, 7), 442 (M^.+^-NHNHCOCH_3_, 5), 437 (M^.+^+2-Br, 100), 435 (M^.+^-Br, 84) and 355 (M^.+^-Br_2_, 1); Anal. Calc. for C_21_H_17_Br_2_N_5_O (515.20): C, 48.96; H, 3.33; N, 13.59%, found: C, 49.00; H, 3.36; N, 13.60%.

*7-Acetylhydrazino-3,4-dibromo-5-(p-methoxyphenyl)-2-phenylpyrazolo[1,5-c]-pyrimidine* (**8c**). Yield 75%, 0.40 g, mp 182–183 °C; IR (υ_max_, cm^−1^): 3269 (NH), 1668 (C=O), 1612 (pyrazole ring C=N), 1535 (pyrimidine ring C=N), and 1443 (C=C); ^1^H NMR (CDCl_3_, δ_H_, ppm): 2.47 (s, 3H, COCH_3_), 3.79 (s, 3H, OCH_3_), 7.48–7.62 (m, 3H, aromatic-H), 7.91 (d, 2H, aromatic-H), 8.04 (d, 2H, aromatic-H), 8.08 (d, 2H, aromatic-H), 10.05 (s, 1H, exchangeable NH) and 10.10 (s, 1H, exchangeable NHCO); MS, *m/z* (%) = 535(M^.+^+4, 1), 533 (M^.+^+2, 6), 531 (M^.+^, 10), 489 (M^.+^-COCH_2_, 6), 475 (M^.+^-NCOCH_2_, 13), 459 (M^.+^-NNHCOCH_3_, 23), 453 (M^.+^+2-Br, 73), 451 (M^.+^-Br, 61) and 370 (M^.+^+1-Br_2_, 1); Anal. Calc. for C_21_H_17_Br_2_N_5_O_2_ (531.20): C, 47.48; H, 3.23; N, 13.18%, found: C, 47.50; H, 3.26; N, 13.20%.

*7-Acetylhydrazino-5-(p-chlorophenyl)-3,4-dibromo-2-phenylpyrazolo[1,5-c]-pyrimidine* (**8d**). Yield 74%, 0.40 g, mp 226–227 °C; IR (υ_max_, cm^−1^): 3267 (NH), 1664 (C=O), 1620 (pyrazole ring C=N), 1570 (pyrimidine ring C=N), and 1437 (C=C); ^1^H NMR (DMSO-*d_6_*, δ_H_, ppm): 2.47 (s, 3H, COCH_3_), 7.49–7.57 (m, 5H, aromatic-H), 8.04 (d, 2H, aromatic-H), 8.15 (d, 2H, aromatic-H), 10.06 (s, 1H, exchangeable NH) and 10.17 (s, 1H, exchangeable NHCO); MS, *m/z* (%) = 538 (M^.+^+2, 2), 535 (M^.+^-1, 4), 493 (M^.+^- COCH_3_, 4), 463 (M^.+^-NHNHCOCH_3_, 3), 458 (M^.+^+2-Br, 20), 456 (M^.+^-Br, 100) and 376 (M^.+^-Br_2_, 1); Anal. Calc. for C_20_H_14_Br_2_ClN_5_O (535.62): C, 44.85; H, 2.63; N, 13.08%, found: C, 44.81; H, 2.60; N, 13.11%.

#### 3.2.8. 7-Acetylhydrazino-5-aryl-3-iodo-2--phenylpyrazolo[1,5-*c*]pyrimidines **9a-d**

A solution of iodine monochloride (0.2 g, 1.2 mmol) in acetic acid (10 mL) was gradually added to a suspension of 7-acetylhydrazino-5-aryl-2-phenylpyrazolo[1,5-*c*]pyrimidines **7a-d** (1 mmol) in acetic acid (10 mL) with stirring for 3 h at room temperature. The reaction mixture was then poured onto crushed ice and the precipitated 7-acetylhydrazino-5-aryl-3-iodo-2-phenylpyrazolo[1,5-*c*]pyrimidines **9a-d** were filtered, washed with water, dried and crystallized from EtOH as colorless needles.

*7-Acetylhydrazino-3-iodo-2,5-diphenylpyrazolo[1,5-c]pyrimidine* (**9a**). Yield 85%, 0.40 g, mp 229–230 °C; IR (υ_max_, cm^−1^): 3433 (NH), 1680 (C=O), 1618 (pyrazole ring C=N), 1549 (pyrimidine ring C=N), and 1438 (C=C); ^1^H NMR (DMSO-*d_6_*, δ_H_, ppm): 2.05 (s, 3H, COCH_3_), 7.29–7.80 (m, 10H, aromatic-H), 7.48 (s, 1H, pyrimidine-H), and 8.06 (s, 2H, exchangeable NHCO and exchangeable NH); MS, *m/z* (%) = 469 (M^.+^, 2), 451 (M^.+^- H_2_O, 4), 437 (M^.+^- CH_4_O, 2), 343 (M^.+^+1-I, 5), 300 (M^.+^- I-CH_2_CO, 32) and 385 (M^.+^ - CH_3_INO, 100); Anal. Calc. for C_20_H_16_IN_5_O (469.28): C, 51.19; H, 3.44; N, 14.92%, found: C, 51.20; H, 3.40; N, 14.88%.

*7-Acetylhydrazino-3-iodo-2-phenyl-5-p-tolylpyrazolo[1,5-c]pyrimidine* (**9b**). Yield 83%, 0.40 g, mp 204–205 °C; IR (υ_max_, cm^−1^): 3273 (NH), 1661 (C=O), 1610 (pyrazole ring C=N), 1564 (pyrimidine ring C=N), and 1433 (C=C); ^1^H NMR (CDCl_3_, δ_H_, ppm): 2.26 (s, 3H, CH_3_), 2.42 (s, 3H, COCH_3_), 7.18 (s, 1H, pyrimidine-H), 7.28–7.30 (m, 3H, aromatic-H), 7.49–7.52 (m, 4H, aromatic-H), 7.84 (d, 2H, aromatic-H), 7.97 (s, 1H, exchangeable NH), and 7.99 (s, 1H, exchangeable NHCO); MS, *m/z* (%) = 484 (M^.+^+1, 83), 441 (M^.+^-COCH_2_, 34), 426 (M^.+^-NCOCH_3_, 12), 412 (M^.+^-NNCOCH_3_, 12), 410 (M^.+^ -NHNHCOCH_3_, 1) and 357 (M^.+^+1- I, 16); Anal. Calc. for C_21_H_18_IN_5_O (483.30): C, 52.19; H, 3.75; N, 14.49%, found: C, 52.50; H, 3.79; N, 14.52%.

*7-Acetylhydrazino-3-iodo-5-(p-methoxyphenyl)-2-phenylpyrazolo[1,5-c]-pyrimidine* (**9c**). Yield 80%, 0.40 g, mp 124–125 °C; IR (υ_max_, cm^−1^): 3290 (NH), 1711 (C=O), 1610 (pyrazole ring C=N), 1560 (pyrimidine ring C=N), and 1448 (C=C); ^1^H NMR (CDCl_3_, δ_H_, ppm): 2.16 (s, 3H, COCH_3_), 3.87 (s, 3H, OCH_3_), 6.99–7.49 (m, 7H, aromatic-H), 7.69 (s, 1H, pyrimidine-H), 8.01 (d, 2H, aromatic-H) and 9.28 (s, 2H, exchangeable NH and exchangeable NHCO); MS, *m/z* (%) = 484 (M^.+^-CH_3_, 1), 343 (M^.+^- NCOCH_2_, 3), 373 (M^.+^+1-I, 6) and 372 (M^.+^- I, 1); Anal. Calc. for C_21_H_18_IN_5_O_2_ (499.30): C, 50.52; H, 3.63; N, 14.03%. Found: C, 50.55; H, 3.59; N, 14.06%.

*7-Acetylhydrazino-5-(p-chlorophenyl)-3-iodo-2-phenylpyrazolo[1,5-c]pyrimidine* (**9d**). Yield 80%, 0.40 g, mp 207–208 °C; IR (υ_max_, cm^−1^): 3273 (NH), 1662 (C=O), 1616 (pyrazole ring C=N), 1566 (pyrimidine ring C=N), and 1439 (C=C); ^1^H NMR (DMSO-*d_6_*, δ_H_, ppm): 2.01 (s, 3H, COCH_3_), 7.14 (d, 2H, aromatic-H), 7.40 (s, 1H, pyrimidine-H), 7.49–7.54 (m, 3H, aromatic-H), 7.68 (d, 1H, aromatic-H), 7.80 (d, 1H, aromatic-H), 7.98 (d, 2H, aromatic-H),. 10.00 (s, 1H, exchangeable NH), and 10.16 (s, 1H, exchangeable NHCO); MS, *m/z* (%) = 506 (M^.+^+2, 45), 504 (M^.+^, 100), 463 (M^.+^+2-COCH_3_, 22), 461 (M^.+^-COCH_3_, 57), 446 (M^.+^-NHCOCH_3_, 15), 432 (M^.+^ -NNHCOCH_3_, 17), 379 (M^.+^+2-I, 10) and 377 (M^.+^-I, 24); Anal. Calc. for C_20_H_15_ClIN_5_O (503.72): C, 47.69; H, 3.00; N, 13.90%, found: C, 47.72; H, 2.99; N, 13.92%.

#### 3.2.9. 7-Acetylhydrazino-5-aryl-3-nitro-2-phenylpyrazolo[1,5-*c*]pyrimidines **10a-d**

A mixture of nitric acid (d 1.41, 1 mL) and sulfuric acid (d 1.84, 1 mL) in glacial acetic acid (10 mL) was gradually added to a suspension of 7-acetylhydrazino-5-aryl-2-phenylpyrazolo[1,5- *c*]pyrimidines **7a-d** (1 mmol) in glacial acetic acid (10 mL) with stirring for 3 h at room temperature. The reaction mixture was then poured onto cold water with stirring and the yellow precipitated solid were filtered, washed with cold water, dried and crystallized from EtOH to give the title compounds **10a-d** as yellow needles.

*7-Acetylhydrazino-3-nitro-2,5-diphenylpyrazolo[1,5-c]pyrimidine* (**10a**). Yield 77%, 0.30 g, mp 191–192 °C; IR (υ_max_, cm^−1^): 3436 (NH), 1743 (C=O), 1633 (pyrazole ring C=N), 1551 (pyrimidine ring C=N), and 1462 (C=C); ^1^H NMR (CDCl_3_, δ_H_, ppm): 1.90 (s, 3H, COCH_3_), 7.37 (s, 1H, pyrimidine-H), 7.44–7.68 (m, 6H, aromatic-H), 7.97 (d, 2H, aromatic-H), 8.03 (d, 2H, aromatic-H), 8.46 (s, 1H, exchangeable NH), and 9.26 (s, 1H, exchangeable NHCO); MS, *m/z* (%) = 389 (M^.+^+1, 4), 360 (M^.+^- CO, 7), 346 (M^.+^-COCH_2_, 2), 332 (M^.+^-NCOCH_2_, 100), 316 (M^.+^-NNHCOCH_3_, 4), 302 (M^.+^-C_3_H_6_N_2_O, 17), 286 (M^.+^-C_2_H_2_N_2_O_3_, 10) and 258 (M^.+^-C_3_H_4_N_3_O_3_, 25); Anal. Calc. for C_20_H_16_N_6_O_3_ (388.38): C, 61.85; H, 4.15; N, 21.64%, found: C, 61.89; H, 4.12; N, 21.60%.

*7-Acetylhydrazino-3-nitro-2-phenyl-5-p-tolylpyrazolo[1,5-c]pyrimidine* (**10b**). Yield 75%, 0.30 g, mp 194–195 °C; IR (υ_max_, cm^−1^): 3371 (NH), 1745 (C=O), 1601 (pyrazole ring C=N), 1533 (pyrimidine ring C=N), and 1443 (C=C); ^1^H NMR (CDCl_3_, δ_H_, ppm): 1.96 (s, 3H, CH_3_), 2.44 (s, 3H, COCH_3_), 7.30–7.71 (m, 10H, aromatic-H and pyrimidine-H) and 8.44 (s, 2H, exchangeable NH and exchangeable NHCO); MS, *m/z* (%) = 403 (M^.+^+1, 2), 402 (M^.+^, 3), 401 (M^.+^-1, 16), 374 (M^.+^-CO, 8) and 346 (M^.+^-C_2_H_2_NO, 6); Anal. Calc. for C_21_H_18_N_6_O_3_ (402.41): C, 62.68; H, 4.51; N, 20.88%, found: C, 62.71; H, 4.50; N, 20.91%.

*7-Acetylhydrazino-5-(p-methoxyphenyl)-3-nitro-2-phenylpyrazolo[1,5-c]-pyrimidine* (**10c**). Yield 71%, 0.30 g, mp 132–133 °C; IR (υ_max_, cm^−1^): 3464 (NH), 1747 (C=O), 1696 (pyrazole ring C=N), 1531 (pyrimidine ring C=N), and 1454 (C=C); ^1^H NMR (CDCl_3_, δ_H_, ppm): 1.81 (s, 3H, COCH_3_), 3.89 (s, 3H OCH_3_), 7.00–7.71 (m, 10H, aromatic-H and pyrimidine-H) and 8.43 (s, 2H, exchangeable NH and exchangeable NHCO); MS, *m/z* (%) = 420 (M^.+^+2, 1), 358 (M^.+^-C_2_H_6_NO, 1), 334 (M^.+^ -C_3_H_4_N_2_O, 1) and 300 (M^.+^-C_2_H_4_N_3_O_3_, 3); Anal. Calc. for C_21_H_18_N_6_O_4_ (418.41): C, 60.28; H, 4.34; N, 20.09%, found: C, 60.31; H, 4.32; N, 20.13%.

*7-Acetylhydrazino-5-(p-chlorophenyl)-3-nitro-2-phenylpyrazolo[1,5-c]-pyrimidine* (**10d**). Yield 71%, 0.30 g, mp 174–175 °C; IR (υ_max_, cm^−1^): 3371 (NH), 1749 (C=O), 1597 (pyrazole ring C=N), 1533 (pyrimidine ring C=N), and 1443 (C=C); ^1^H NMR (CDCl_3_, δ_H_, ppm): 2.13 (s, 3H, COCH_3_), 7.34–7.73 (m, 10H, aromatic-H and pyrimidine-H) and 8.45 (s, 2H, exchangeable NH and exchangeable NHCO); MS, *m/z* (%) = 423 (M^.+^, 1), 381 (M^.+^-COCH_2_, 1), 366 (M^.+^- NCOCH_3_, 1), 324 (M^.+^-C_3_H_5_N_3_O, 1) and 300 (M^.+^-C_6_H_5_NO_2_, 2); Anal. Calc. for C_20_H_15_ClN_6_O_3_ (422.82): C, 56.81; H, 3.58; N, 19.88%, found: C, 56.77; H, 3.61; N, 19.85%.

#### 3.2.10. 7-Triacetylhydrazino-5-aryl-2-phenylpyrazolo[1,5-*c*]pyrimidines **11a-d**

A suspension of 5-aryl-7-hydrazino-2-phenylpyrazolo[1,5-*c*]pyrimidines **1a-d** (1 mmol) in acetic anhydride (5 mL) was heated under reflux for 1 h and the mixture was cooled and poured onto crushed ice. The product that separated out was filtered off, washed with water and then dried. It was crystallized from EtOH to give the title compounds **11a-d** as colorlees needles.

*7-Triacetylhydrazino-2,5-diphenylpyrazolo[1,5-c]pyrimidine* (**11a**). Yield 83%, 0.35 g, mp 179–180 °C; IR (υ_max_, cm^−1^): 1736 (C=O), 1623 (pyrazole ring C=N), 1542 (pyrimidine ring C=N) and 1458 (C=C); ^1^H NMR (DMSO-*d_6_*, δ_H_, ppm): 2.45 (s,3H, COCH_3_), 2.48 (s, 3H, COCH_3_), 2.50 (s, 3H, COCH_3_), 7.28 (s, 1H, pyrazole-H), 7.44 (t, 2H, aromatic-H), 7.50 (t, 4H, aromatic-H), 7.99 (d, 2H, aromatic-H) , 8.05 (d, 2H, aromatic-H) and 8.24 (s, 1H, pyrimidine-H); MS, *m/z* (%) = 428 (M^.+^+1, 15), 385 (M^.+^-CH_2_CO, 15), 343 (M^.+^-2 CH_2_CO, 100), 301 (M^.+^-3 CH_2_CO, 65), 286 (M^.+^- C_6_H_6_NO_2_, 24), 272 (M^.+^-C_6_H_6_N_2_O_2_, 88) and 270 (M^.+^-C_6_H_8_N_2_O_2_, 26); Anal. Calc. for C_24_H_21_N_5_O_3_ (427.46): C, 67.44; H, 4.95; N, 16.38%, found: C, 67.40; H, 5.00; N, 16.40%.

*7-Triacetylhydrazino-2-phenyl-5-p-tolylpyrazolo[1,5-c]pyrimidine* (**11b**). Yield 80%, 0.35 g, mp 194–195 °C; IR (υ_max_, cm^−1^): 1726 (C=O), 1622 (pyrazole ring C=N), 1520 (pyrimidine ring C=N) and 1450 (C=C); ^1^H NMR (CDCl_3_, δ_H_, ppm): 2.17(s, 3H, CH_3_), 2.42 (s,3H, COCH_3_), 2.54 (s, 3H, COCH_3_), 2.56 (s, 3H, COCH_3_),6.90 (s, 1H, pyrazole-H), 7.28 (d, 2H, aromatic-H), 7.42–7.50 (m, 3H, aromatic-H), 7.72 (s, 1H, pyrimidine-H), 7.87 (d, 2H, aromatic-H) and 7.95 (d, 2H, aromatic-H); MS, *m/z* (%) = 443 (M^.+^+2, 2), 441 (M^.+^, 10), 399 (M^.+^-COCH_2_, 11), 357 (M^.+^-2COCH_2_, 100), 315 (M^.+^-3COCH_2_, 44) and 300 (M^.+^-C_6_H_7_NO_3_, 21); Anal. Calc. for C_25_H_23_N_5_O_3_ (441.48): C, 68.01; H, 5.25; N, 15.86%, found: C, 68.12; H, 5.22; N, 15.81%.

*7-Triacetylhydrazino-5-p-methoxyphenyl-2-phenylpyrazolo[1,5-c]pyrimidine* (**11c**). Yield 76%, 0.35 g, mp 132–133 °C; IR (υ_max_, cm^−1^): 1720 (C=O), 1614 (pyrazole ring C=N), 1510 (pyrimidine ring C=N), and 1448 (C=C); ^1^H NMR (CDCl_3_, δ_H_, ppm): 2.53(s, 9H, 3COCH_3_), 3.86 (s, 3H, OCH_3_), 6.87 (s, 1H, pyrazole-H), 6.98 (d, 2H, aromatic-H), 7.41–7.49 (m, 3H, aromatic-H), 7.65 (s, 1H, pyrimidine- H) and 7.91–7.95 (m, 4H, aromatic-H); MS, *m/z* (%) = 459 (M^.+^+2, 2), 457 (M^.+^, 17), 415 (M^.+^- COCH_2_, 19), 373 (M^.+^-2COCH_2_, 100), 331 (M^.+^-3COCH_2_, 39), 316 (M^.+^-C_6_H_7_NO_3_, 17) and 302 (M^.+^-C_7_H_9_NO_3_, 41); Anal. Calc. for C_25_H_23_N_5_O_4_ (457.48): C, 65.63; H, 5.07; N, 15.31%, found: C, 65.51; H, 5.05; N, 15.28%.

*7-Triacetylhydrazino-5-p-chlorophenyl-2-phenylpyrazolo[1,5-c]pyrimidine* (**11d**). Yield 76%, 0.35 g, mp 174–175 °C; IR (υ_max_, cm^−1^): 1722 (C=O), 1616 (pyrazole ring C=N), 1533 (pyrimidine ring C=N), and 1443 (C=C); ^1^H NMR (CDCl_3_, δ_H_, ppm): 2.52(s, 3H, COCH_3_), 2.56 (s, 6H, 2COCH_3_), 6.91 (s, 1H, pyrazole-H), 7.41–7.49 (m, 5H, aromatic-H), 7.70 (s, 1H, pyrimidine-H), 7.88 (d, 2H, aromatic-H) and 7.93 (d, 2H, aromatic-H); MS, *m/z* (%) = 464 (M^.+^+2, 1), 462 (M^.+^, 5), 420 (M^.+^-COCH_2_, 8), 378 (M^.+^-2COCH_2_, 58), 336 (M^.+^-3COCH_2_, 30), 320 (M^.+^-C_6_H_8_NO_3_, 13) and 306 (M^.+^-C_6_H_8_N_2_O_3_, 24); Anal. Calc. for C_24_H_20_ClN_5_O_3_ (461.90): C, 62.41; H, 4.36; N, 15.16%, found: C, 62.40; H, 4.32; N, 15.20%.

## 4. Conclusions

In summary, the strategy for constructing the target compounds started by 5-aryl-7-hydrazino-2- phenylpyrazolo[1,5-*c*]pyrimidines **1a-d** where the hydrazine moiety can be readily heterocyclized with one-carbon inserting agents to give the triazole ring fused to the pyrazolopyrimidine skeleton has been successfully demonstrated.
